# Development and validation of cardiac diffusion weighted magnetic resonance imaging for the diagnosis of myocardial injury in small animal models

**DOI:** 10.1038/s41598-024-52746-5

**Published:** 2024-02-12

**Authors:** Chul Hwan Park, Pan Ki Kim, Yoonjung Kim, Tae Hoon Kim, Yoo Jin Hong, Eunkyung Ahn, Yoon Jin Cha, Byoung Wook Choi

**Affiliations:** 1 Department of Radiology and the Research Institute of Radiological Science, Gangnam Severance Hospital, Yonsei University College of Medicine, Seoul, Republic of Korea; 2 Department of Radiology and the Research Institute of Radiological Science, Severance Hospital, Yonsei University College of Medicine, Seoul, Republic of Korea; 3 Department of Laboratory Medicine, Gangnam Severance Hospital Yonsei University College of Medicine, Seoul, Republic of Korea; 4 Department of Pathology, Gangnam Severance Hospital, Yonsei University College of Medicine, 211 Eonju-ro, Gangnam-gu, Seoul, 06273 Republic of Korea

**Keywords:** Cardiology, Cardiovascular diseases

## Abstract

Cardiac diffusion weighted-magnetic resonance imaging (DWI) has slowly developed due to its technical difficulties. However, this limitation could be overcome by advanced techniques, including a stimulated echo technique and a gradient moment nulling technique. This study aimed to develop and validate a high-order DWI sequence, using echo-planar imaging (EPI) and second-order motion-compensated (M012) diffusion gradient applied to cardiac imaging in small-sized animals with fast heart and respiratory rates, and to investigate the feasibility of cardiac DWI, diagnosing acute myocardial injury in isoproterenol-induced myocardial injury rat models. The M012 diffusion gradient sequence was designed for diffusion tensor imaging of the rat myocardium and validated in the polyvinylpyrrolidone phantom. Following sequence optimization, 23 rats with isoproterenol-induced acute myocardial injury and five healthy control rats underwent cardiac MRI, including cine imaging, T1 mapping, and DWI. Diffusion gradient was applied using a 9.4-T MRI scanner (Bruker, BioSpec 94/20, gradient amplitude = 440 mT/m, maximum slew rate = 3440 T/m/s) with double gating (electrocardiogram and respiratory gating). Troponin I was used as a serum biomarker for myocardial injury. Histopathologic examination of the heart was subsequently performed. The developed DWI sequence using EPI and M012 provided the interpretable images of rat hearts. The apparent diffusion coefficient (ADC) values were significantly higher in rats with acute myocardial injury than in the control group (1.847 ± 0.326 * 10^–3^ mm^2^/s vs. 1.578 ± 0.144 * 10^–3^ mm^2^/s, *P* < 0.001). Troponin I levels were increased in the blood samples of rats with acute myocardial injury (*P* < 0.001). Histopathologic examinations detected myocardial damage and subendocardial fibrosis in rats with acute myocardial injury. The newly developed DWI technique has the ability to detect myocardial injury in small animal models, representing high ADC values on the myocardium with isoproterenol-induced injury.

## Introduction

The water molecules move consistently according to the thermal energy^1^. Depending on the microenvironment surrounding water molecules, the diffusion of water molecules can create a diffusion contrast^2^.

In the 1960s, diffusion-weighted imaging (DWI) using magnetic resonance techniques was emerged to evaluate the random motion of water molecules^3^. Initially, DWI technique has been applied to a variety of intra-cranial diseases^4^. With technical advancements in magnetic resonance imaging (MRI), such as high gradient amplitude parallel imaging and echo-planar imaging (EPI), the DWI application has been expanded to the body, including the lungs and liver^2,5^. Diffusion MRI of the heart was initially described in 1994^6^. Using diffusion parameters, it is possible to understand the fiber architecture in healthy and diseased hearts; DWI potentially evaluates and characterizes the cardiac pathology in response to injury or disease through changes in water diffusivity of the myocardium. However, it still has unresolved technical limitations that hinder its clinical usage. The main technical limitations of cardiac DWI are as follows: (1) motion artifacts from breathing and cardiac pulsations, (2) bulk movement of blood flow, (3) short T2 relaxation time of the myocardium, (4) insufficient spatial resolution, (5) insufficient gradient power, (6) long image acquisition time, and (7) image distortion due to strong magnetic susceptibility artifact^7–9^.

Since the periodic movement of the heart and lung has a significantly larger bulk motion than the Brownian motion of the water molecules, it is relatively difficult to perform the commonly used Stejskal-Tanner method in cardiac DWI (2). However, this limitation could be overcome by diffusion imaging using advanced techniques, including a stimulated echo technique and a gradient moment nulling technique in the heart^10^.

### Purposes of this study

This study aimed to develop and validate the high-order diffusion-weighted MR sequence using an EPI and a second-order motion-compensated (M012) diffusion gradient, which could be applied to the heart of small-sized animals with fast heartbeats and respiration, and to investigate the possibility of cardiac DWI in the in vivo rat heart for the early diagnosis of acute myocardial injury.

## Results

### DWI sequence development

**Figure 1:**
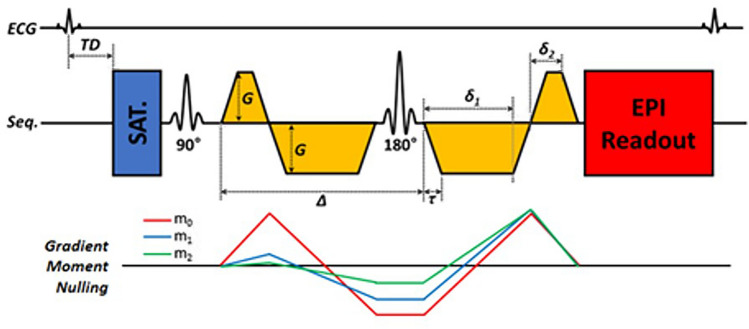
Sequence for the cardiac diffusion-weighed imaging with second-order motion compensation.

Figure 1 demonstrates the final version of the developed second-order motion-compensated DWI sequence designed for the diffusion tensor imaging of the in vivo rat myocardium.

This high-order cardiac diffusion-weighted imaging sequence was developed with a velocity and acceleration compensable diffusion gradient to suppress cardiac and respiratory motion-induced artifacts based on the EPI readout.

### Phantom test

The ADC values of the polyvinylpyrrolidone (PVP) concentrate from 0 to 50% w/v were 1.99, 1.83, 1.66, 1.53, 1.30, and 1.28 * 10^−3^ mm^2^/s on the DTI-EPI with M0. The ADC values of the PVP concentrate from 0 to 50% were 2.21, 2.04, 1.84, 1.67, 1.41, and 1.37 * 10^−3 ^mm^2^/sec on the DTI EPI with M012. The correlation coefficient between DTI-EPI with M0 and DTI-EPI with M012 was 1.0 (Supplementary Fig. S1).

### Validation and parameter optimization of second-order motion-compensated diffusion-weighted imaging

**Figure 2:**
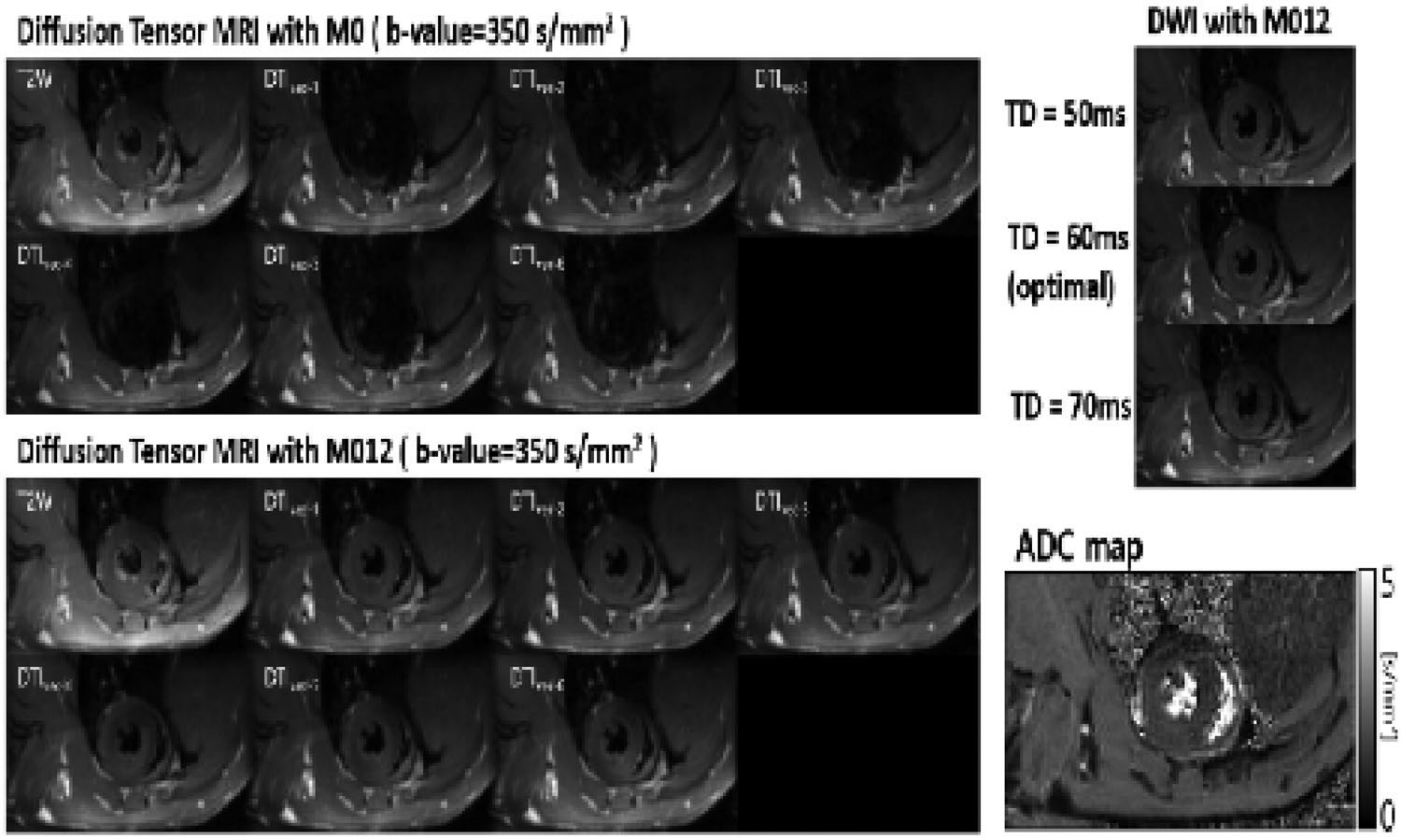
Results of validation and parameter optimization of second-ordermotion-compensated diffusion-weighed imaging.

Figure 2 shows a DTI-EPI (b-value = 350 s/mm^2^) images with M0 and M012 in a healthy rat. For DTIs with only M0, the heart was not observed except when b-value was zero. However, in DTIs with M012, the myocardium was observed in all diffusion directions. DTI images were observed without distortion when the trigger delay time was 60 ms (end-systolic phase). High-quality ADC maps were obtained from the in vivo rats using the DTI of M012.

### Validation with chronic infarction model

On the late gadolinium enhancement (LGE) images, myocardial infarction was delineated with a high signal intensity in the left anterior descending artery (LAD) territory. The ADC value of the infarcted myocardium on the ADC map was 1.735 ± 0.060 * 10^−3 ^mm^2^/s. The ADC value of the remote myocardium was 1.484 ± 0.072 * 10^−3 ^mm^2^/s. The ADC value of the infarcted myocardium was significantly higher than that of the remote myocardium (*P* < 0.001) (Supplementary Fig. S2).

### Animal study with acute myocardial injury model

The mean heart rate of the control group was 255 ± 21 bpm. The heart rates of groups A and B were 325 ± 57 bpm and 290 ± 28 bpm, respectively. The heart rate of group A was significantly higher than that of the control group or group B (*P* = 0.023). The left ventricular ejection fractions (%) of the control group, group A, and group B were 73.1 ± 2.5, 75.9 ± 2.6, and 75.7 ± 3.4, respectively (*P* = 0.10). The mean ECV fractions of the LV myocardium (%) were 14.5 ± 1.6, 18.4 ± 6.5, and 14.1 ± 4.1 in the control group, group A, and group B, respectively (*P* = 0.133) (Table 1).

**Table 1:** Basic characteristics of the controls and myocardial injury models.

	Control (n = 5)	Group A (n = 11)	Group B (n = 12)	*P*-value
Heart rate (bpm)	255 ± 21	325 ± 57*	290 ± 28	0.023
LVEF (%)	73.1 ± 2.5	75.9 ± 2.6	75.7 ± 3.4	0.10
ECV fraction (%)	14.5 ± 1.6	18.4 ± 6.5	14.1 ± 4.1	0.133

*bpm* beats per minute, *LVEF* left ventricle ejection fraction, *ECV* extracellular volume.

*P < 0.05, compared with the control group or group B.

**Figure 3:**
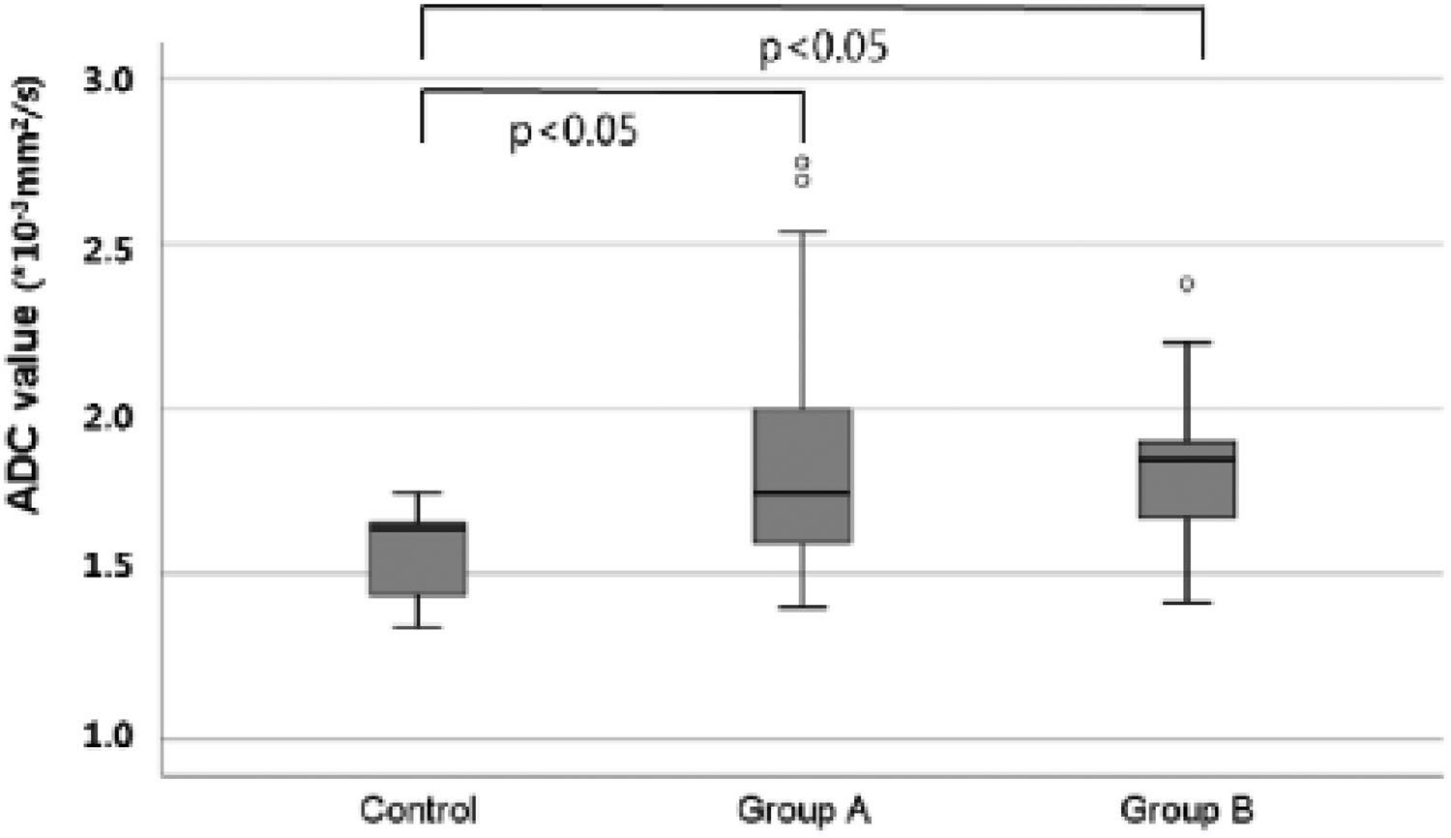
Differences of apparent diffusion coefficient values between the control group and myocardial injury group.

**Figure 4:**
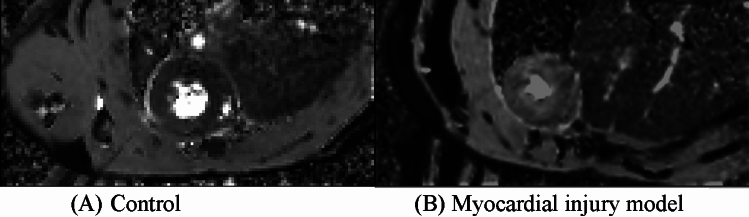
Comparison of apparent diffusion coefficient maps between the control group and acute myocardial injury model. The myocardial apparent diffusion coefficient (ADC) value of the acute myocardial model (**B**) is significantly higher than that of the control group (**A**).

The myocardial ADC value of the control group was 1.578 ± 0.144 * 10^–3^ mm^2^/s. The ADC value of acute myocardial injury models (groups A and B, n = 23) was 1.847 ± 0.326 * 10^–3^ mm^2^/s and significantly higher than that of the control group (*P* < 0.001). The myocardial ADC values of groups A (n = 11) and B (n = 12) were 1.873 ± 0.401 * 10^–3^ mm^2^/s and 1.820 ± 0.232 * 10^–3^ mm^2^/s, and significantly higher than that of the control group (*P* = 0.002). However, the ADC value of group A was not significantly different from that of group B (*P* = 0.999) (Figs. 3, 4).

### Serum chemistry: troponin I

**Figure 5:**
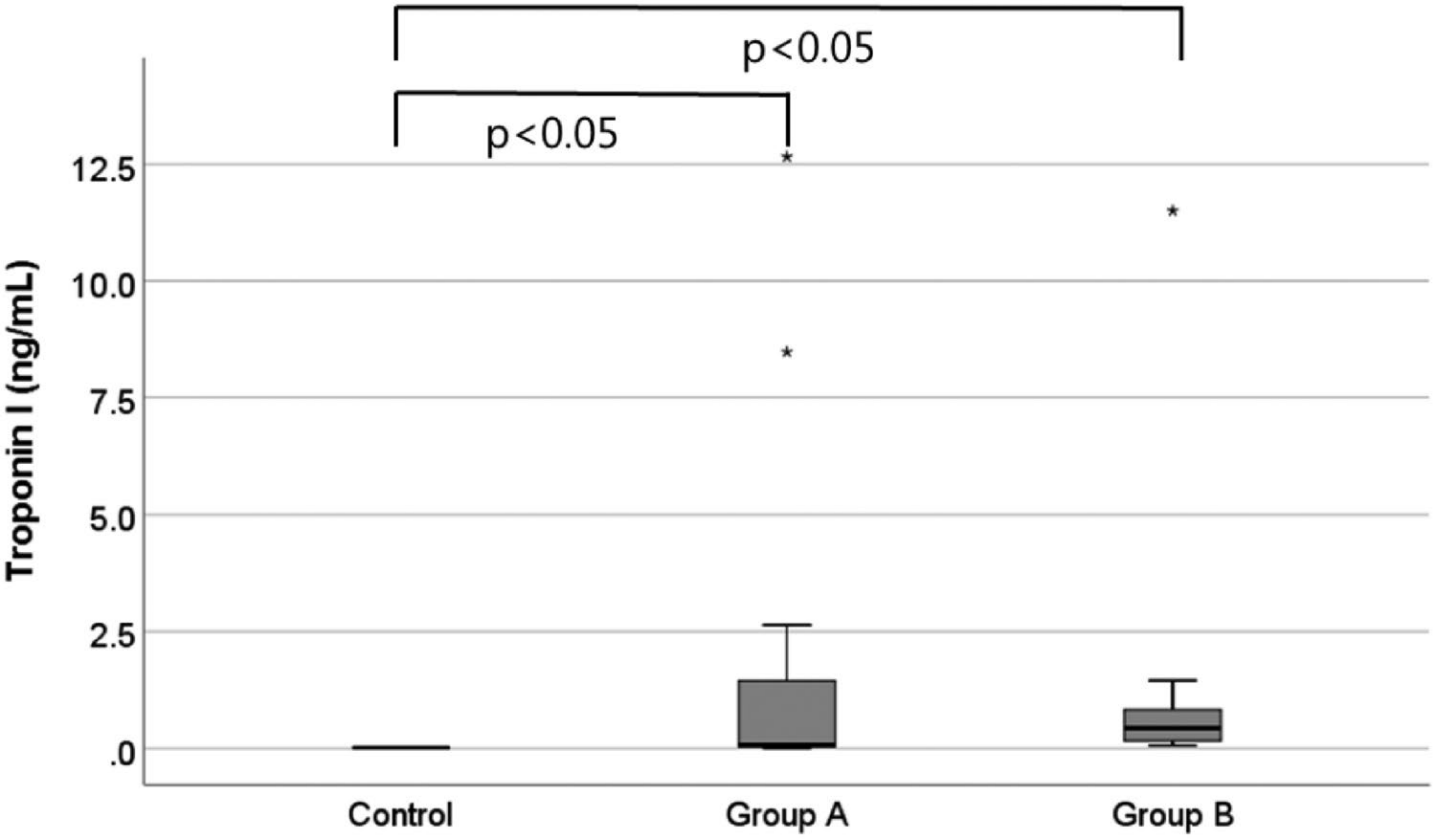
Differences of serum cardiac troponin I values between the control group and myocardial injury group.

The cTnI level in the control group was lower than 0.0023 ng/mL. The cTnI levels in groups A and B were 2.214 ± 4.30 ng/mL and 1.481 ± 3.35 ng/mL, respectively. The cTnI level was significantly higher in the acute myocardial injury model than in the control group (*P* < 0.001) (Fig. 5).

### Histological findings

**Figure 6:**
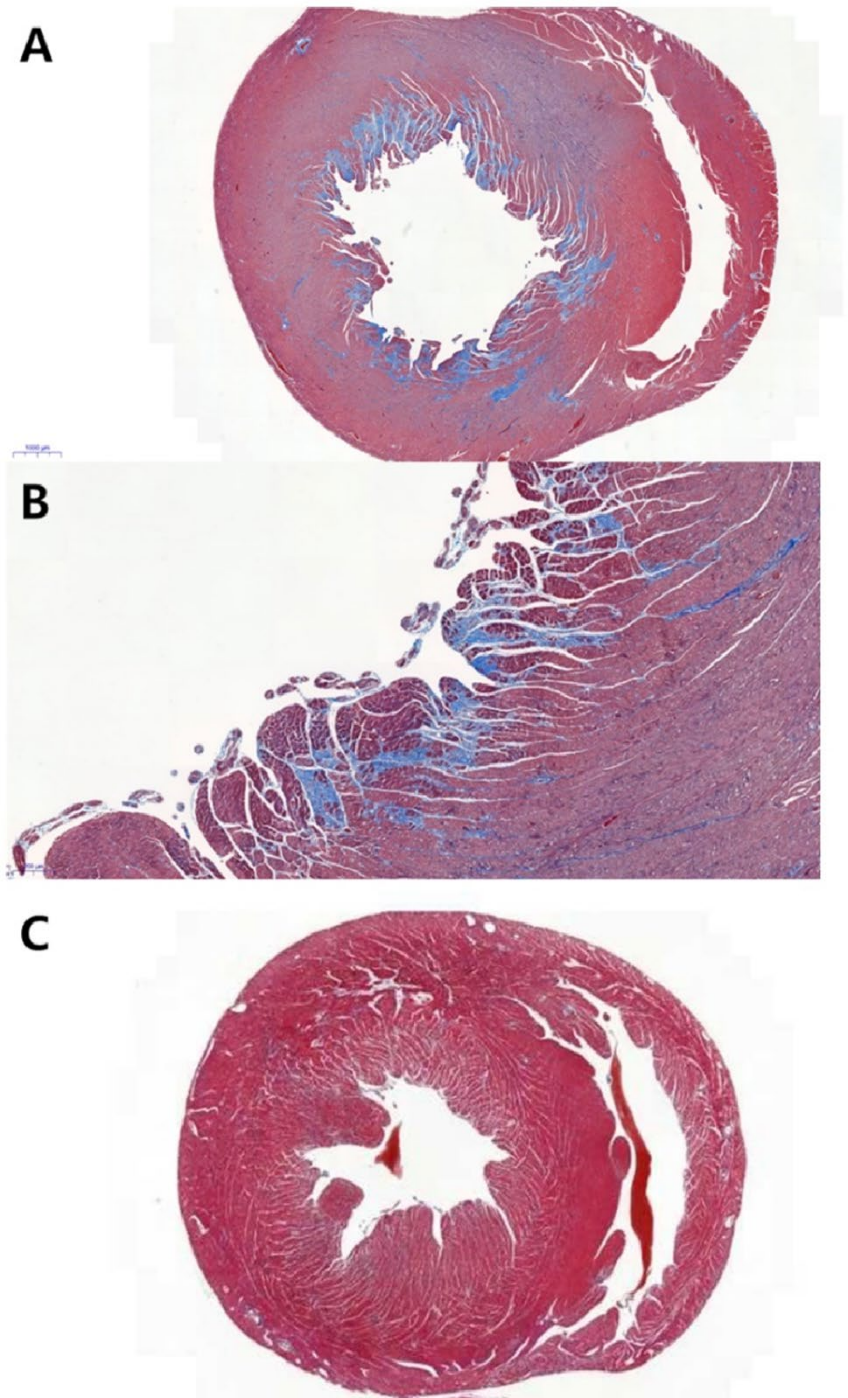
Histopathologic finding of a myocardial injury model. (**A**,**B**) Group A in acute myocardial injury model (Masson’s trichrome staining; A: no magnification, B: magnification, × 5) reveals circumferential subendocardial fibrosis. On the contrary, (**C**) group B reveals (Masson’s trichrome staining; no magnification) no subendocardial fibrosis.

Compared to the control group, myocardial injury group showed a more diffuse interstitial edema with increased intercellular spaces on the H&E stain (Supplementary Fig. S3). In group A (n = 11), three rats (27.2%) revealed circumferential subendocardial fibrosis in the left ventricle on the Masson’s trichrome staining (Fig. 6). None of the rats revealed abnormal myocardial fibrosis in group B (n = 12). The control group revealed that gross abnormalities were not observed, such as myocardial edema or myocardial injuries.

## Discussion

In this study, a cardiac DWI with EPI and M012 was developed and validated in small-sized animals with fast cardiac and respiratory cycles. As a result, we were able to obtain high-quality ADC maps that could detect myocardial injury, representing high ADC values on the myocardium with isoproterenol (ISO)-induced injury.

MRI is a useful imaging technique for observing diffusion. Molecular motion in tissues can be divided into bulk flow, isotropic diffusion, and restricted diffusion^11^. Bulk motion refers to the net transport of molecules from one place to another, such as blood flow or tissue movement, and can be represented as flow or velocity. On the contrary, diffusion is not a movement of molecules to another location but an irregular movement within a specific region. Isotopic diffusion, such as pure water, is easily dispersed in the spin phase, but if diffusion is restricted, the phase dispersion of the spin is small, resulting in a higher signal in the image^2,12^. Based on these principles, DWI is actively used in various organs, including the brain, liver, and prostate. However, cardiac DWI is considerably challenging due to technical difficulties including motion artifacts from breathing and cardiac pulsations, bulk movement of blood flow, short T2 relaxation time of the myocardium, insufficient spatial resolution, insufficient gradient power, long image acquisition time, and image distortion due to the strong magnetic susceptibility of artifacts^1,11,13^. However, there are technical breakthroughs to perform cardiac DWI. Stimulated echo (STE) is a type of echo that originates from three radiofrequency (RF) pulses. Since the diffusion-encoded STE sequence is performed over two consecutive heartbeats, several RF pulses, and long diffusion times, it can produce a mixing time during a substantial diffusion sensitization^6,8^. It uses low gradient strength and short gradient duration even in low gradient MR system, resulting in a higher b-value effect. The STE diffusion imaging is currently the most realistic heart diffusion imaging in clinical MR system^8^. However, this STE diffusion imaging is susceptible to irregular heart rhythm, and the signal-to-noise ratio (SNR) decreases by more than half compared to the SE methods^14^. Gradient moment nulling is one of the motion artifact suppression techniques that modifies the gradient waveform to suppress cardiac motion-related artifacts resulting in an additional phase due to imperfect gradient refocusing^10,15^. This can be achieved by changing the shape and number of gradient waveforms to null the gradient moments ($m_{0}$, $m_{1}$, $m_{2}$, and $m_{3}$). The Stejskal-Tanner method is inherently $m_{0}$-nulled, and other gradient moments need not be considered, assuming that the object is motionless (or is not moving). The higher-order motion compensated ($m_{0}$, $m_{1}$, $m_{2}$-nulled) diffusion gradients show promising results for in vivo motion-robust cardiac diffusion MR^13,16^. This high compensation increases the TE and reduces the SNR, but enables reliable diffusion imaging even in unpredictable physiological movements without significantly increasing scan time^17,18^.

In this study, we developed cardiac DWI sequence with EPI and M012 to enable the diffusion tensor imaging in the rat hearts. The developed sequence was validated through phantom studies and animal studies. The feasibility of the developed cardiac DWI was evaluated with acute myocardial injury models using ISO injection. ISO is a sympathomimetic agent that stimulates beta-adrenergic receptors. The drug accelerates sinoatrial nodes and enhances atrioventricular node conduction^19^. An ISO injection could induce myocardial fibrosis, infarction, necrosis, mitochondrial change, left ventricular dilatation, and myocardial hypertrophy^20^. Animal models revealed a significant increase in troponin I levels on blood examination and mild subendocardial fibrosis on pathologic examination representing myocardial injury. Myocardial injury induces myocardial edema and fibrosis. During early edema, water enters the myocardial cells due to a decrease in the function of the pumps on the cellular membrane, known as intracellular edema^21^. If myocardial damage persists, the cellular membranes may rupture, causing intracellular materials to emerge out of the cell and cause extracellular edema^22^. The native T1 values are useful for detecting myocardial edema^23,24^. However, T1 values can be affected by various conditions^25^. The LGE imaging and ECV fraction are useful in detecting myocardial fibrosis^23,24^. For LGE imaging and ECV fraction, injection of contrast medium is mandatory^23–25^, and the exact co-registration of native T1 images and post T1 images is important for delicate analysis for the ECV fraction^26^. On the contrary, cardiac DWI can detect diffusion changes in the myocardium, the main characteristic of various myocardial diseases, without the need for contrast material injection^16^. Myocardial edema increases the diffusion capacity^2^, and Moulin et al.^27^ reported a quantitative ADC map representing myocardial edema in acute infarction. On the contrary, fibrosis could lower water diffusion^28^.

DWI could characterize various myocardial changes quantitatively by using various parameters, including ADC, mean diffusivity fractional anisotropy (FA), and helix angle (HA)^29^. Architectural change with a reorientation of myocardial fiber could be evaluated in the infarcted and remote myocardium using HA^29,30^. Recently, Das et al.^31^ reported FA is an independent indicator of long-term adverse remodeling after ST-segment elevation MI. DWI is a feasible method that evaluates myocardial fibrosis in HCM^1,32^. In DCM, reduced reorientation of myofiber aggregates and steeper HA are associated with LV remodeling and reduced strain^33^. Detection of increased myocardial water content using DWI can be helpful in the diagnosis and monitoring of acute myocarditis or stress-induced cardiomyopathy^34^. DWI is considered a promising noninvasive method for cardiac diseases, including ischemic cardiomyopathy and non-ischemic cardiomyopathy, by providing a deeper understating of cardiac microstructure, function, and disease-related changes without using a gadolinium-based contrast material^35^.

This study has several limitations. First, this is a single-center animal study with a small sample size using a 9.4-T MR system. To evaluate the human heart with a clinically available MR system, further sequence modification and validation are required. Second, in the phantom study without motion, DTI-EPI with M012 showed a perfect correlation with DTI-EPI with M0. However, the absolute values were slightly higher in DTI-EPI with M012. This systemic bias should be addressed before clinical application. In addition, even though three-step validation was done, microstructural imaging was not carried out during the validation process. Third, in this study, only the ADC value was evaluated despite the presence of various diffusion-related parameters, including FA and HA. However, 6-direction acquisition helped to reduce motion and EPI artifact. Fourth, DTI-EPI with M012 did not compare to the conventional DWI without high-order compensation because conventional DWI could not provide interpretable ADC maps in beating hearts. Fifth, the lack of quantitative assessment of histologic changes hinders the evaluation of the clinical usefulness of DWI.

## Conclusion

In conclusion, we demonstrated the feasibility of DTI-EPI with M012 in small-sized animals with fast cardiac and respiratory cycles. The accuracy of the DTI-EPI images with M012 was confirmed by comparing the ADC maps using PVP samples of various concentrations. This high-order motion compensated DWI imaging technique has the ability to diagnose acute myocardial injury, representing high ADC values on the myocardium with isoproterenol-induced injury in small animal models.

## Materials and methods

### Ethical statements

This study was approved by the Institutional Animal Care and Use Committee in our institution, and the procedures were performed in accordance with the National Institutes of Health guidelines and this study is reported in accordance with ARRIVE guidelines.

### DWI sequence development

M012 diffusion gradient was designed for the diffusion tensor imaging (DTI) of the rat myocardium. Diffusion gradient was applied using a 9.4-T MRI scanner (Bruker, BioSpec 94/20; gradient amplitude, 440 mT/m; maximum slew rate, 3440 T/m/s). To minimize physiological motion artifacts from heartbeats and respiration, double gating (electrocardiogram [ECG] + respiratory gating) was applied. MRI parameters were as follows: TR/TE, 2500 ms/20 ms; field of view (FOV), 50 × 25 mm; matrix, 128 × 64; excitation/refocusing thickness, 2/4 mm; b-value, 350 s/mm^2^; diffusion direction, 6; and EPI segmentation, 2, with 4 channels receiving RF coil. The trigger delay was set to obtain DTI during the end-systolic cardiac phase for the minimum cardiac motion. The trigger delay was defined as the time from the ECG's R-wave to the first RF pulse of the M012 sequence. The scan time was reduced by applying a saturation band to areas other than the FOV.

### Phantom test

To verify the performance of M012 diffusion gradient, six samples with various concentrations of PVP (Sigma-Aldrich, K value, 29–32) were prepared. PVP solutions in the range of 0–50% w/v were prepared in distilled water. The diffusion tensor images with general Stejskal-Tanner (M0) diffusion gradient and M012 diffusion gradient were obtained using the diffusion phantom, and subsequently, ADC values were calculated. The ADC results were compared for two methods.

### Validation with normal rats and chronic myocardial infarction model

For the validation of developed M012 sequence, DWI was performed on the three commercially available chronic myocardial infarction models (Central Lab. Animal Inc. Slc:Wistar) and three normal rats (adult male Sprague–Dawley rats with weight range of 300–500 g) in vivo. With normal rats, diffusion tensor images with general M0 diffusion gradient and M012 diffusion gradient were obtained on various readout times, and subsequently, image qualities were compared. With chronic myocardial infarction model, diffusion tensor images with M012 diffusion gradient were obtained; subsequently, ADC values were compared between the infarcted myocardium and remote myocardium.

### Animal modeling of isoproterenol-induced myocardial injury model

**Figure 7:**
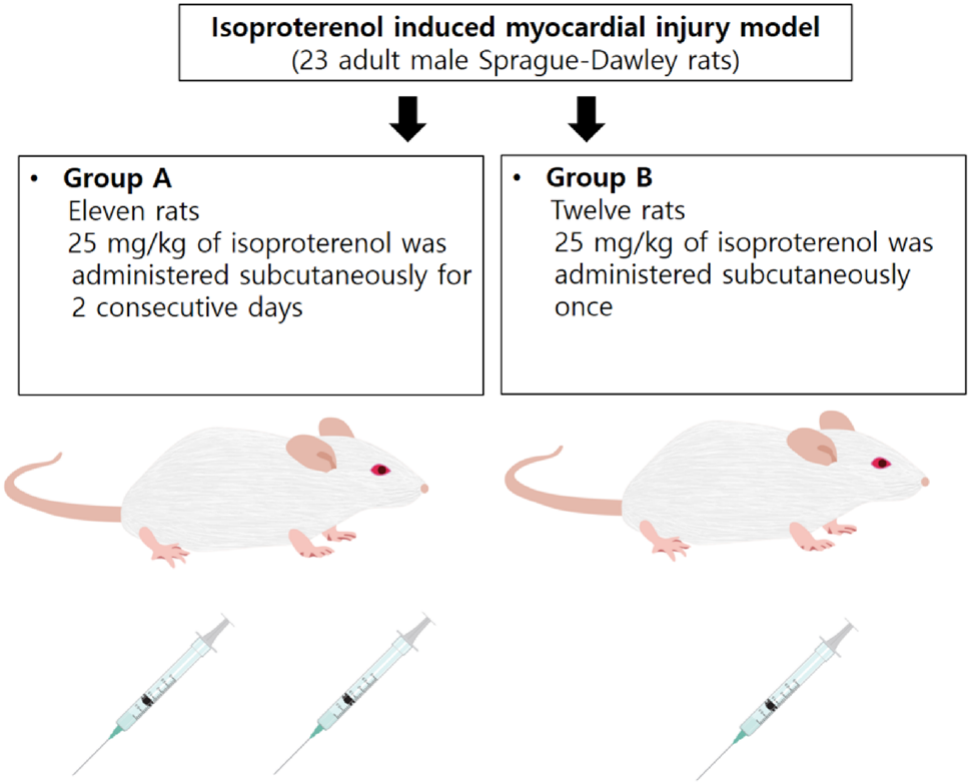
Isoproterenol-induced myocardial injury model.

Twenty-three adult male Sprague–Dawley rats (weight range, 300–500 g) were assigned to an ISO-induced myocardial injury model and classified into two groups. Eleven rats were assigned into group A, and 25 mg/kg of ISO (Sigma Chemical Co, Poole, Dorset, UK) was administered subcutaneously for 2 consecutive days. Twelve rats were assigned into group B, and 25 mg/kg of ISO was administered once subcutaneously (Fig. 7).

### Control group

Five adult male Sprague–Dawley rats (weight range, 300–500 g) were assigned as the control group.

### Cardiac MR imaging

All cardiac MRI examinations were performed using a 9.4-T MR scanner (Bruker BioSpec; Bruker Biospin, Billerica, Mass) with an 86-mm volume coil and a four-channel phased-array surface coil. After localizing the heart using a fast low-angle shot sequence, cine images were acquired in the two- and four-chamber and short-axis planesusing a fast low-angle shot sequence with electrocardiography and respiratory gating (Supplementary Fig. S4A,B). The parameters for cine imaging were as follows: 4.0/1.16; flip angle, 15°; 25 phases; section thickness, 2 mm; two signals acquired; acquisition matrix, 128 × 128; and FOV, 50 × 50 mm. After the acquisition of cine imaging, the native T1 mapping images were acquired using a saturation recovery Look-Locker sequence (Supplementary Fig. S5A,B). After the native T1 mapping was obtained, diffusion-weighted imaging was performed with a second-order motion compensated diffusion gradient sequence. Double gating (ECG + respiratory gating) was applied to minimize physiological motion artifacts from heartbeats and respiration.

The diffusion MRI parameters were as follows: TR/TE = 2500 ms/20 ms, FOV = 50 × 25 mm, matrix = 128 × 64, excitation/refocusing thickness = 2/4 mm, b-value = 350 s/mm^2^, diffusion direction = 6, and EPI segmentation = 2, with 4 channels receiving RF coil. The trigger delay was set so that DTI was obtained at the systolic cardiac phase for the minimum cardiac motion. The contrast-enhanced T1 mapping images were obtained to calculate the extracellular volume (ECV) fraction after the injection of a contrast medium. Blood sampling was performed after MRI to measure the hematocrit (Hct) levels. During MRI, the rats were sedated with an inhalation anesthetic agent (a mixture of oxygen and isoflurane), and gentle pressure was applied to the torso of the rats to minimize motion artifacts caused by respiration.

### Cardiac MR analyses

Two expert radiologists with more than 10 years of experience in cardiovascular image interpretation independently analyzed the images.

A. Left ventricular function

All MR cine images were transferred to the cvi42 image analysis software (Circle Cardiovascular Imaging Inc., Calgary, AB, Canada). The left ventricular (LV) function was assessed on the short-axis cine MR images using the Simpson’s method. The endocardial and epicardial borders of the LV walls were semiautomatically delineated on end-diastolic and end-systolic images. The LV end-diastolic and LV end-systolic volumes were automatically measured, and the LV ejection fraction (%) was calculated as follows: ([LV end-diastolic volume—LV end-systolic volume]/LV end-diastolic volume) *100 (Supplementary Fig. S4C).

B. T1 values and extracellular volume fraction

All T1 mapping images were transferred to the cvi42 image analysis software. The endocardial and epicardial borders of the LV walls were semiautomatically delineated on the pre- and post-contrast T1 images obtained in the short-axis plane of the mid-LV level. T1 values were semiautomatically measured from the mid-LV myocardium using the American Heart Association 17-segment model (Supplementary Fig. S5C,D). A round > 5 mm^2^ region of interest, avoiding papillary muscles, was drawn in the LV cavity. The myocardial ECV was also automatically calculated using the Hct value, the native and post-contrast T1 values of the LV myocardium, and the blood cavity^36^.

C. Diffusion parameters

**Figure 8:**
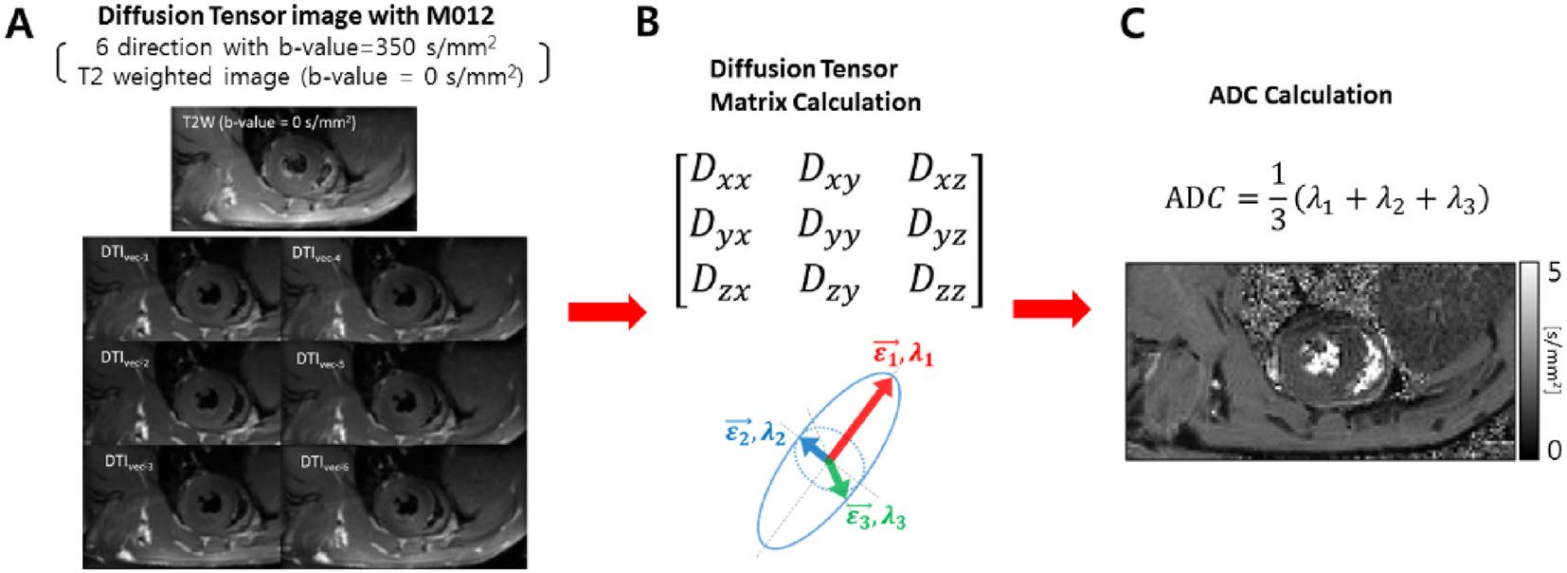
Diffusion-weighted imaging: Acquisition and analysis. (**A**) Seven images including 6-directional diffusion-weighted image with b-value 350 s/mm^2^ and a T2-weighted image with b-value 0 s/mm^2^ are obtained from beating rat hearts. (**B**) By using the obtained seven images, diffusion tensor matrix is created, and subsequently, eigenvalues and eigenvectors are evaluated from diffusion ellipsoid. Then, (**C**) the ADC is calculated by solving the diffusion tensor matrix.

The signal attenuation of the diffusion-weighted image depends on multiple factors, such as $$S = S_{0} \cdot {\text{exp}}\left( { - b \cdot D} \right)$$, where $$S_{0}$$ is a signal without diffusion gradient(like T2-weighted image), $$b$$ is a degree of diffusion weighting (*b*-value), and *D* is a diffusion coefficient^5^. The b -value is defined as $$b = \gamma^{2} G^{2} \delta^{2} \left( {\Delta - {\raise0.7ex\hbox{$\delta $} \!\mathord{\left/ {\vphantom {\delta 3}}\right.\kern-0pt} \!\lower0.7ex\hbox{$3$}}} \right)$$, where $$\gamma$$ is the gyromagnetic ratio; $$G$$ and $$\delta$$ are the strength and duration of the diffusion gradient, respectively; and $$\Delta$$ is the time interval between the two diffusion gradients^37^. All apparent diffusion coefficient (ADC) values were calculated from seven images, including a 6-directional diffusion-weighted images with b-value 350 s/mm^2^ and a T2-weighted image with b-value 0 s/mm^2^ by a homemade program of the MATLAB software. The ADC equation is presented as Fig. 8, where $$\lambda$$ is the eigenvalue that is output by solving the diffusion tensor matrix^1,38^.

### Blood sample collection and analysis

After sacrificing the rats and extracting their hearts, the blood was collected from the aorta, and separated plasma was stored at − 80 °C. The cardiac troponin I (cTnI) levels were measured as a biomarker of myocardial injury using the hsTnI assay (Beckman Coulter, Inc. Brea, CA, USA). The hsTnI detection limit was 2.3 pg/mL.

### Histological examination

After cardiac MRI, the rats were sacrificed for a histological examination using a carbon dioxide chamber. After euthanasia, the heart was immediately extracted. Each heart was fixed in 10% phosphate-buffered paraformaldehyde. After a week of fixation, the heart was sectioned serially along the short-axis plane. The section of the heart at the mid-ventricular level where the papillary muscles were visible was embedded in the paraffin. The two slices of the section were stained with hematoxylin and eosin (H&E) and Masson’s trichrome staining by an experienced pathologist blinded to the MR results. H&E staining was used to evaluate myocardial injury, while Masson’s trichrome staining was used to evaluate myocardial fibrosis in extracellular space.

### Statistical analyses

All continuous data were expressed as means ± standard deviations, and categorical variables were presented as numbers or percentages. The Shapiro–Wilk test was performed to evaluate the data distribution. The significance of differences in functional parameters, ECV fractions, and ADC value levels were evaluated using student’s *t-*test, Mann–Whitney U test, analysis of variance test, or Kruskal–Wallis test between the controls and the acute myocardial injury groups. All statistical analyses were performed using the Statistical Package for the Social Sciences (SPSS 23, Chicago, IL, U.S.A.), and a *P* value < 0.05 was considered statistically significant.

### Supplementary Information

Supplementary Figures.

## Data Availability

The datasets used and/or analyzed during the current study available from the corresponding author on reasonable request.
