# Supplementary material for: Development and validation of cardiac diffusion weighted magnetic resonance imaging for the diagnosis of myocardial injury in small animal models
Source: Sci Rep. 2024 Feb 12;14:3552. doi: 10.1038/s41598-024-52746-5 (PMC10861543; doi:10.1038/s41598-024-52746-5)
Supplement: Supplementary file 1 — Supplementary Figures. [file 41598_2024_52746_MOESM1_ESM.docx]

**Supplementary figure 1. Results of phantom study for the validation of the developed high-order motion-compensated diffusion-weighted imaging sequence**


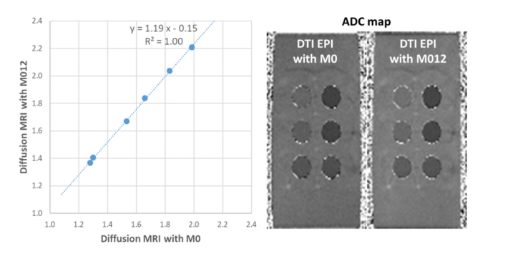


**Supplementary figure 2. Validation with chronic infarction model**


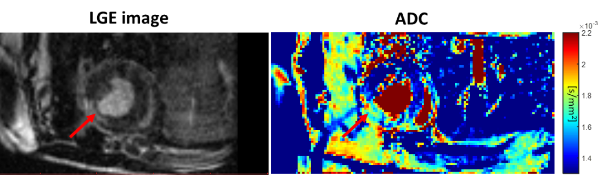


**(A)                             (B)**

On the LGE images, myocardial infarction is delineated with a high signal intensity in the LAD territory (A). The ADC value of the infarcted myocardium on the ADC map (B) is significantly higher than that of the remote myocardium.

**Supplementary figure 3. Comparison of histologic findings (hematoxylin and eosin stain) between the control group and myocardial injury group**

Histological images (H&E stain *400) of (A) control and (B) myocardial injury model: The myocardium of the myocardial injury model shows a wider intercellular space compared to the control, suggesting interstitial edema.


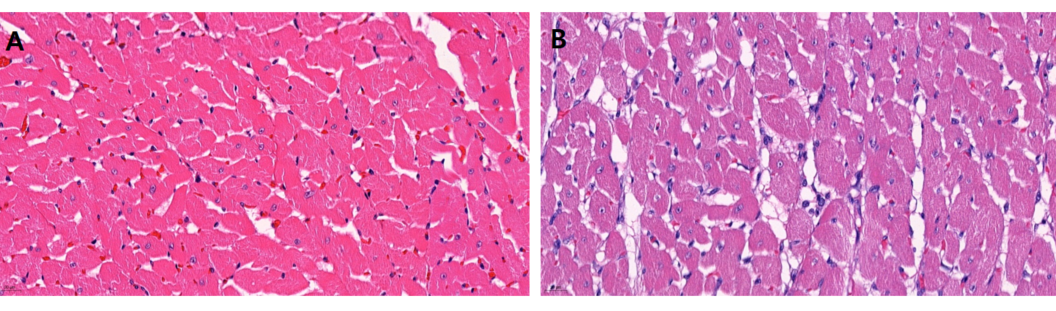


**Supplementary figure 4. Cine imaging: Acquisition and analysis**


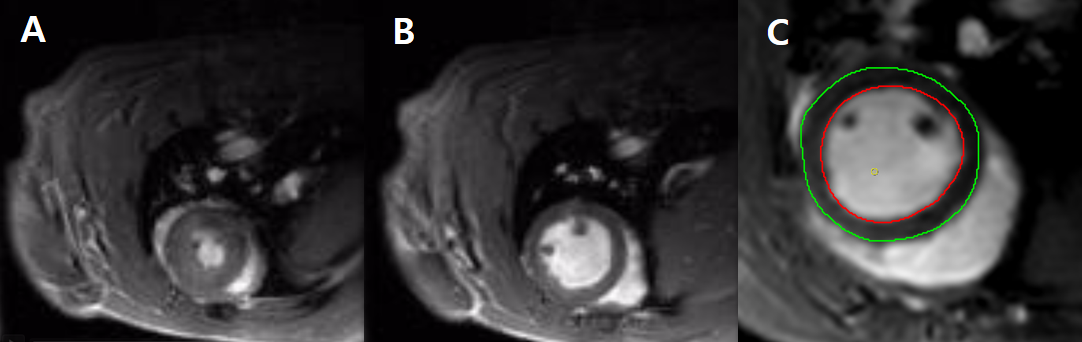


(A, B) Cine images (A: end-systolic phase, B: end-diastolic phase) obtained from a rat model of isoproterenol-induced myocardial injury show sufficient image quality for left ventricular (LV) function analysis. (C) By using cardiac MR dedicated software, LV systolic function is evaluated.

**Supplementary figure 5. T1 map imaging: Acquisition and analysis**


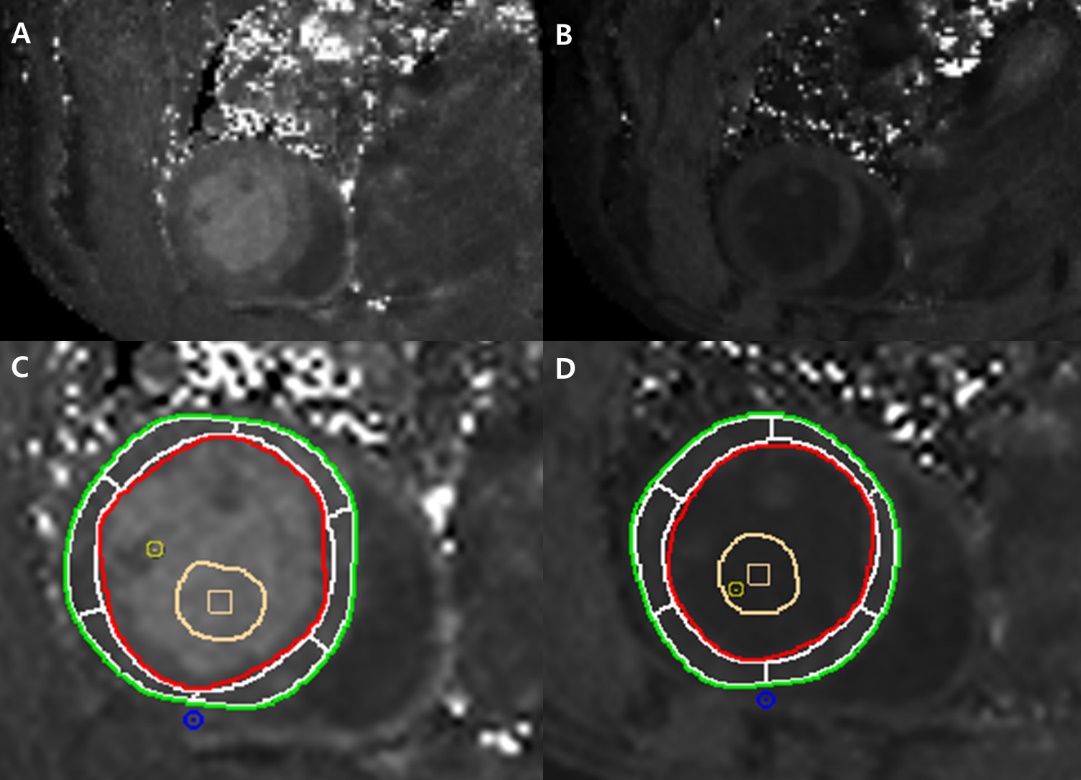


(A) Native T1 image and (B) post T1 image are obtained using a saturation recovery Look-Locker sequence in the short-axis plane. (C, D) By using cardiac MR dedicated software, T1 values are semiautomatically measured from the mid-left ventricular myocardium.
